# HyBloc: Localization in Sensor Networks with Adverse Anchor Placement

**DOI:** 10.3390/s90100253

**Published:** 2009-01-08

**Authors:** King-Yip Cheng, King-Shan Lui, Vincent Tam

**Affiliations:** Department of Electrical and Electronic Engineering, The University of Hong Kong, Pokfulam Road, Hong Kong; E-Mails: kycheng@eee.hku.hk (K-Y. C); vtam@eee.hku.hk (V. T.)

**Keywords:** localization, wireless sensor networks, anchor placement

## Abstract

To determine the geographical positions of sensors, numerous localization algorithms have been proposed in recent years. The positions of sensors are inferred from the connectivity between sensors and a set of nodes called anchors which know their precise locations. We investigate the effect of adverse placement and density of anchors on the accuracies of different algorithms. We develop an algorithm called *HyBrid Localization* (HyBloc) to provide reliable localization service with a limited number of clustered anchors. HyBloc is distributed in nature with reasonable message overhead. Through simulations, we demonstrate that HyBloc provides more accurate location estimates than some existing distributed algorithms when there are only a few anchors. HyBloc also performs well when anchors are clustered together.

## 1. Introduction

Localization is an important service in mobile computing, robotics and wireless sensor networks. Many wireless sensor network applications become possible only when precise location information of sensors is available, e.g. habitat monitoring, target tracking, location-based routing, etc. However, determining the location of sensors is a non-trivial task especially when taking the large scale of sensor networks into account. Although equipping receivers with GPS (Global Positioning System) enables sensors to obtain precise location information, the costs prohibit the network-wide deployment. Furthermore, the reception of GPS signal is subjected to the underlying environmental conditions. Another possible solution is manually configure each sensor and install sensors at the predetermined positions. This solution becomes inefficient when the network size increases. To enable location service in wireless sensor networks, various localization algorithms [1–9] have been proposed in recent years.

Different algorithms employ various mathematical techniques to determine the positions of sensors, such as multilateration [1, 8], multidimensional scaling (MDS) [3, 4], and mathematical programming [2, 6, 10], etc. Basically, all existing algorithms can be classified into *centralized algorithms* and *distributed algorithms*. In a centralized algorithm, a powerful processing node collects the global topology information and then calculates the locations for all nodes in the networks. On the contrary, in a distributed algorithm, a non-anchor node identifies its own location based on the locality information. However, most algorithms assume the existence of anchors with knowledge about their physical locations by equipping receivers with GPS, manual configuration or other means. Although various algorithms use the location information of anchors differently, the number and placement of anchors affect the accuracies of localization algorithms to a certain extent. Substantial amount of anchors are required to maintain the accuracy for distributed algorithms based on multilateration. Studies [11–13] show that the placement of anchors also affect the performance of multilateration-based algorithms. The performance is better when anchors are uniformly distributed along the perimeter of the network. Centralized algorithms are less susceptible to the density and placement of anchors but they are less scalable at the same time. A centralized server is required to collect all the data, determine the solution and distribute the position estimates to the other sensors.

In this paper, we first study the performances of several representative algorithms when anchors are limited and clustered. To the best of our knowledge, our paper is the first one that addresses this issue. The results show that none of them perform well. We then develop our solution, a distributed localization algorithm called Hybrid Localization (HyBloc) [14]. HyBloc can work with very few anchors and gives comparable results to those centralized algorithms. Furthermore, it is less susceptible to the effect of adverse anchor placement. HyBloc performs well even when anchors are clustered together. This property is especially useful when anchors cannot be spread across the network. For example, if sensors are dropped to monitor an area where GPS signal reception is poor and manual configuration is infeasible, such as a place deep inside a canyon or understory of a dense tropical rain forest, only a few anchors can localize themselves by GPS and are clustered at the place where GPS signal can be well-received. Other sensors have to rely on these anchors to determine their own locations.

The paper is organized as follows. In Section II, we discuss some of the previous works of localization in wireless sensor networks. In Section III, we give the details of our proposed algorithms. Section IV presents the simulation results and the comparison with existing algorithms. Lastly, we conclude our work in Section V.

## 2. Related Works

Localization algorithms can be classified as distributed or centralized algorithms. Most distributed localization algorithms employ multilateration in which nodes localize by themselves. On the other hand, centralized algorithms can be further classified into other sub-categories such as multidimensional scaling (MDS) or mathematical programming.

### 2.1. Distributed Algorithms

Niculescu *et al.* [1] proposed a pioneering distributed localization algorithm called Ad Hoc Positioning System (APS). APS uses triangulation which is similar to the Global Positioning System (GPS). The mechanism of APS becomes a model for many other localization algorithms devised later. A sensor node first estimates the distances from at least 3 (4 in 3-D) anchors whose location information is accurate and known. A system of non-linear equations relating the distance estimates, the positions of the sensors and positions of anchors is established. The system is approximated as a linear system and is solved iteratively by each sensor. There are three propagation methods to disseminate the distance information, namely DV-Hop, DV-Distance and Euclidean.

DV-Hop and DV-Distance adopt the conventional distance-vector exchange methods. Each anchor node broadcasts a message to all nodes in the network. The message carries the location of the broadcasting anchor. In DV-Hop, the message keeps the accumulated hop count along the path it has traversed, while in DV-Distance, it keeps the accumulated path distance. The distance between a sensor and an anchor is estimated by the hop count or the path distance. In DV-Hop, it is estimated by multiplying the hop count and the average hop size. The average hop size is obtained by dividing the geographical distance by the hop count between anchors. Though the absence of ranging makes DV-Hop immune to measurement noise, the coarse estimation of distance gives a poor performance if the variance of the hop size is large. To eliminate this adverse effect, the shortest-path distance is used as the distance estimate in DV-Distance. The last propagation method, Euclidean, propagates the true Euclidean distance. For sensors which are not connected to an anchor, it has to seek help from other sensors to determine the true Euclidean distance. Therefore, a high density of anchors is needed to localize all the nodes in the network. a high density of anchors is needed. To allow 90% of the nodes to localize, the percentage of anchors required is as high as 40%.

Savarese *et al.* [7] proposed another propagation-based algorithm called Hop-TERRAIN. The algorithm is similar to DV-Hop which uses both the hop count and average hop size to estimate the distance between a sensor and an anchor. Furthermore, the position estimates is also obtained by solving a system of linear equations. After obtaining an initial estimation, each sensor refines its estimation through successive triangulation with its one-hop neighbours. Since the objective of the first phase of Hop-TERRAIN is to obtain a rough estimation, the estimates are unreliable and hence neighbours suffer from severe error propagation if no anchor can be found in its one-hop neighbours. To mitigate the error propagation, different weights are assigned to different equations. The weights depend on the confidence level of the location information of neighbours. Location information of anchors has the largest weight as its coordinates are assumed to be exact. Though the introduction of weights gives some improvement, the performance of Hop-TERRAIN depends on both the number and the placement of anchors. If anchors are clustered together, sensors that are far away from the cluster have to refine their estimates by other sensors but not anchors, which would not be very accurate. Savvides *et al.* [5] proposed a similar two-phase algorithm that also consists of a refinement phase. Sensor nodes first obtain a rough estimate by utilizing the bounding box constraints. The solution is refined by applying the Kalman filter.

Costa [15] proposed a distributed MDS algorithm for localization in wireless sensor networks. Each node participates in determining the global solution by a local loss function. Although information exchanges are limited within neighbours only, multiple iterations are required to achieve accurate estimates. It introduces substantial message overheads.

Him *et al.* [8] designed a distributed algorithm for *anisotropic* topologies. In general, a network is isotropic if measurements in all directions are exhibiting the same properties; otherwise, it is anisotropic. The major difficulty encountered with anisotropy is the dynamic relation between the geographical distance and the proximity measured by sensors in different directions. Sensor *A* may have equal path distances from sensors *B* and *C* but the actual physical distances between *A,B* and *A,C* can differ a lot. The path of *AB* may detour around a hole while the path of *AC* is a straight path. This is very common in networks with irregular shape, such as a C-shape network as shown in Figure 5. To cope with this situation, Him *et al.* devised a *proximity-distance map* (PDM) to capture the relation in different directions. Anchors derive an optimal linear transformation collaboratively to map the precise geographical information and the proximities between anchors. The map is sent to each sensor. The mechanism of PDM-based localization system is similar to APS except the proximities collected by each sensor are transformed by the proximity-distance map instead of corrected by the correction factor before multi-lateration is performed. The algorithm works well when anchors are distributed uniformly across the network. When anchors are clustered together, the proximity-distance map is no longer reliable. The relation between proximity and geographical distance in the region without any anchor is not characterized by the PDM.

Aspnes *et al.* [16] analysed the localization problem in a theoretical way by the graph rigidity theory. On top of this, Goldenberg [9] *et al.* proposed a localization algorithm called Sweeps for sparse networks. Sweeps is an iterative trilateration algorithm in which nodes are incrementally localized from an initial set of sensors.

### 2.2. Centralized algorithms

Doherty *et al.* [10] proposed a centralized algorithm by formulating the localization problem as a convex optimization problem. Various convex constraints are formulated by collecting connectivity information between sensors. The problem is solved by a centralized solver. The quality of the solution greatly depends on the density and placement of anchors. The major weakness of Doherty's formulation is the ignorance of *bounding out constraints*. Since the problem is formulated as a convex optimization problem, no bounding out constraint can be included in the problem formulation and therefore all bounding out constraints are dropped out. Thus the performance of Doherty's formulation depends on the placement of the anchors. It is better to place anchors around the boundary of the networks so that sensors fall within the convex hull of the anchors. If sensors are beyond the convex hull formed by anchors, the positions estimated will collapse into the convex hull which gives poor accuracy.

Biswas *et al.* [6] alleviated the placement problem by formulating the localization problem as a semidefinite program (SDP) through relaxation. Bounding out constraints are not ignored. Though SDP requires only a few anchors and yields good results, it demands extensive storage and computation when the network size is large. The number of constraints of a problem increases as the connectivity between sensors increases. Although some constraints are redundant and can be removed to reduce the storage requirements, the process of reducing redundant constraints introduces extra computational overheads.

Shang *et al.* [3] proposed a centralized algorithm MDS-MAP which employs multidimensional scaling (MDS) to tackle the localization problem. Multidimensional scaling was originated from psychophysics and is widely used in data analysis. MDS is used to transform data from high dimensional space to points in low (2 to 3) dimensional space so that they can be visualized by human. The algorithm consists of three phases. In the first phase, all-pair-shortest-path distances or hop counts between sensors are collected through distance vector exchange. The path distance provides a rough estimate about the distance between every pair of sensors. In the second phase, classical MDS is applied to yield a relative map in which distances between points are equal or close to the path distance provided. In the final phase, the relative map is transformed to an absolute map through rotation, mirroring and shifting with at least 3 anchors. Since the position information of anchors is only used in determining the linear transformation, MDS-MAP requires comparatively less anchors. Because of the global information and computation required by MDS-MAP, it has to be executed in a centralized fashion. To make it more applicable in sensor networks, Shang *et al.* [4] proposed a distributed version, MDS-MAP(P). The network is divided into different regions whose maps are determined locally. The local maps are merged and transformed into a complete map. The merged map is subsequently transformed into a global map through linear transformation with the location information of anchors. Though MDS-MAP(P) is claimed as a distributed algorithm, a distributed map-merging process has not been addressed yet.

Ahmed *et al.* [17] proposed the SHARP approach which inspired our work. Though SHARP also employs two different localization algorithms in a phased approach, it is designed for relative localization. Nodes positions are localized in the second phase with reference to the coordinate system determined in the first phase. Our proposed algorithm, on the other hand, can obtain absolute coordinates. In addition, we look into the issues of anchor placement.

## 3. Our Hybrid Approach: HyBloc

In this section, we present a distributed localization algorithm named HyBloc which combines two techniques, multidimensional scaling [18] and proximity-distance map [8]. The choice of integrating MDS-MAP and PDM is based on our extensive studies on existing localization algorithms.

### 3.1. Overview

**Figure 1.**
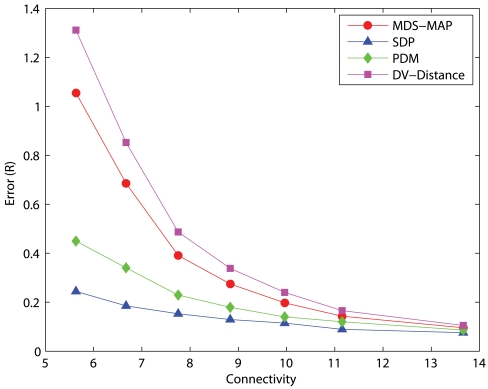
50-node networks, 10 anchors, measurement error=5%.

**Figure 2.**
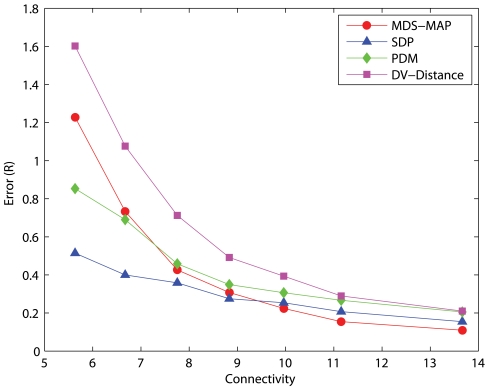
50-node networks, 5 anchors, measurement error=5%.

**Figure 3.**
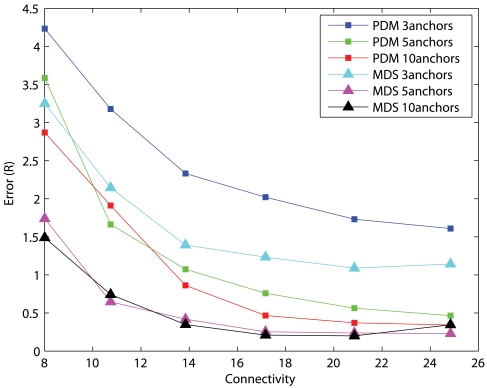
200-node networks, clustered anchors, measurement error=5%.

We have examined the accuracies of representative localization algorithms such as DV-distance of APS, MDS-MAP, SDP, and PDM in networks with 50 uniformly distributed nodes and measurement errors of 5%. The errors presented are normalized to the communication range, *R*, of sensors. The results with 10 anchors randomly distributed across the networks are shown in Figure 1 and the results of 5 anchors are shown in Figure 2. Each data point is the average result of 30 different simulation runs. DV-distance and PDM are distributed algorithms while MDS-MAP and SDP are centralized. Since sensors have limited memory and energy, a distributed algorithm is definitely more suitable than a centralized one. Unfortunately, the results clearly indicate that centralized algorithms outperform distributed ones in terms of precision in most cases. Among the two centralized algorithms MDS-MAP and SDP, although the performance of SDP is better, the high complexity of SDP makes it impractical to be carried out in a sensor. We have tried to solve a problem with 200 nodes using SDP but it was unsolvable with a 3.2GHz PC equipped with 1GB of memory. The problem size was too large that it quickly ran out of the memory available. On the other hand, APS and PDM have similar complexity but the performance of PDM is a lot better. Therefore, we focused our studies in MDS and PDM. Figure 2 shows that PDM cannot perform as good as MDS when there are only a few anchors. We then studied their performance in large networks(200 nodes) where anchors are clustered in a corner. The results are shown in Figure 3.

From the results, we realized that the performance of MDS is less dependent on the number of anchors. Five anchors are enough for a sensor network with 200 uniformly distributed nodes. Other algorithms usually fail to give meaningful results with such a small number of anchors. However, the accuracy of MDS drops significantly when the network topology is irregular in shape [8]. In addition, MDS is inherently a centralized method as it requires all-pair-shortest-path distance estimates between sensors. On the contrary, PDM, as a distributed algorithm, performs very well with an anisotropic sensor network if anchors are well-spread across the network. With wide-spread anchors, proximity-distance map can capture the relationship between the measured proximity and the geographical distance and thus produces an accurate solution. The map fails to capture the global relationship when anchors are clustered together in a local region. Hence, position estimates for nodes that are far away from the clustered anchors become inaccurate. These properties induce our proposed algorithm, HyBloc, a distributed localization algorithm capable of delivering an accurate solution even with a limited number of clustered anchors.

The accuracy of PDM can be improved by having more anchors. That is, if we can artificially “add” some anchors, the performance can be improved. HyBloc employs a hybrid approach in which MDS is used to increase the number of *anchors* in order to extend the anchor coverage of PDM. In the beginning, there are a few nodes that are equipped with GPS receivers and we call these nodes *primary anchors*. In the first phase, a subset of ordinary sensors are selected as *secondary anchors*. Nodes which are neither primary anchors nor secondary one are called normal sensors. The definition of primary and secondary anchors will be employed consistently throughout this paper. The locations of secondary anchors are determined by MDS. The number of secondary anchors is controlled such that MDS can be performed on each selected sensor individually. After the secondary anchors have identified their locations, other ordinary sensors are localized using PDM based on the location information of both the primary and the secondary anchors in the second phase.

### 3.2. The Protocol

The positions of the secondary anchors are based on the primary anchors. It is in fact a small localization problem since the number of secondary anchors is not large. By limiting the amount of secondary anchors, it is justifiable to use MDS for finding the locations of secondary anchors. Our algorithm works as follows. In the first phase, some sensors are selected as secondary anchors and they are localized through MDS. In the second phase, the normal sensors are localized through proximity-distance map. The mapping is derived from the primary and secondary anchors altogether. In the following discussion, we assume that nodes know the number of primary anchors *k_p_* at deployment and the total number of nodes in the network. In case *k_p_* is not known beforehand, in our protocol, each anchor node has to broadcast its location information to all nodes in the network and *k_p_* can be identified. The details of our protocol are given as follows:

1. **Phase Ia: Identification of secondary anchors**
  Due to the different properties of uniform and anisotropic networks, we adopt different ways to assign secondary anchors. Secondary anchors are spread apart in uniform networks but are relatively close in anisotropic networks.
  - Uniform Networks
    In uniform networks, secondary anchors can be more widely spread and can be farther away from the primary anchors than in anisotropic networks. Each primary anchor sends an invitation packet containing its unique ID, two counters initialized to zero and a value *k_s_* controlling the number of secondary anchors, to *one* of its neighbours. One of the counters marks how many secondary anchors have been selected so far while the other marks the hop count that the invitation packet has travelled since the last secondary anchor is selected. Each invitation packet should identify *k_s_* secondary anchors. Normal sensor receiving this packet will perform a Bernoulli trial with a success rate of *p* only if the invitation packet has travelled for at least *x* hops away from the last secondary anchor or primary anchor. The success rate *p* and the *x*-hop restriction roughly controls the separation between secondary anchors so that they will not be clustered together. The values of *p* and *x* can be included in the packet sent by primary anchors or stored in sensor nodes before deployment. If the outcome is true, the normal sensor increments the counter by one and becomes a secondary anchor. The packet will be forwarded to another neighbour until the counter equals to *k_s_*. If a secondary anchor receives a packet originated from other primary anchors, the packet will be forwarded to another node. Thus the total number of primary and secondary anchors will be *k_p_* × (*k_s_* + 1), *k_p_* primary anchors and *k_p_* * *k_s_* secondary anchors.
  - Anisotropic Networks
    In a C-shaped network, path distance is an unreliable Euclidean distance estimate when nodes are far apart. Thus unlike an uniform network, nodes close to the anchors are chosen as secondary anchors. Each primary anchor is responsible to choose *k_s_* secondary anchors. A primary anchor first selects secondary anchors from its direct neighbours. If the number of one hop neighbours is less than *k_s_*, the residue vacancies will be filled up by the two-hop neighbours or three-hop neighbours until *k_s_* secondary anchors are selected. The total number of primary and secondary anchors is also *k_p_* × (*k_s_* + 1).
2. **Phase Ib: Localization of Secondary Anchors**
  In this step, secondary anchors have to acquire the proximity information between every pair of primary and secondary anchors. After sending the invitation packet, each primary anchor sends packets containing its unique ID and coordinates to *all* of its neighbours. The packet also bears a field marking the proximity, i.e. the distance or hop count the packet has travelled. The value is initialized to be zero. Secondary anchors will simply repeat the operation of primary anchors, that is sending out packets with its unique ID but leaving the coordinates field blank.
  Every node (including anchors) receiving a proximity packet from an anchor (either primary or secondary) will store its ID and the proximity value. If a packet from a particular anchor has been received before, the node examines the proximity and checks whether it is larger than the stored proximity. If it is larger than the stored value, the packet will be discarded. Otherwise, the stored value and the proximity field of the packet will be updated and the packet will be forwarded to other neighbours. Thus the stored proximity always reflects the shortest path distance or hop count from a particular anchor.
  After an anchor *x* has discovered its proximities to all anchors, it will send the proximities it has collected to other anchors and wait for other anchors to repeat the same step. When all anchors have distributed the proximities to their counterparts, each anchor knows the proximity information between every pair of anchors. Now, every secondary anchor can determine its location through the classical MDS.
3. **Phase IIa: Anchor Nodes Proximity-Distance Map Calculation**
  After calculating its physical position using MDS, each secondary anchor also knows the position estimates of other secondary anchors since MDS provides a configuration about the primary and secondary anchors. Thus the proximity-distance map **T** among both primary and secondary anchors can be calculated immediately as follows according to [8]:
  Let **P** be the proximity matrix that *p_ij_* is the proximity measure between anchors *i* and *j* where *p_ii_* = 0. The proximity measure can be hop count or cumulative path distance between anchors. Similarly, let **L** be a geographical distance matrix that *l_ij_* is the geographical distance between anchors *i* and *j. l_ij_* can be calculated by utilizing the position estimates obtained from the previous step. **P** and **L** are square matrices with size *m* × *m* where *m* is the total number of primary and secondary anchors (*k_p_* × (*k_s_* + 1)). The PDM **T** is defined as a linear mapping that maps matrix **P** to matrix **L** in which the following error is minimized:
  (1) $$e_{i}={\left\|l_{i}^{T}-t_{i}P\right\|}^{2}$$
  where
  (2) $$l_{i}={\left[l_{i1},\ldots ,l_{im}\right]}^{T},$$
  and *t_i_* is the i-th row of *T*,
  (3) $$T=LP^{T}{\left(PP^{T}\right)}^{-1}$$
  The mapping **T** can be calculated by using *singular value decomposition* (SVD). Every secondary anchor calculates the PDM. The mapping and the position estimates of secondary anchors obtained from the first phase are distributed to the normal nodes nearby.
4. **Phase IIb: Localization of Normal Nodes**
  Each normal sensor node *s* uses the mapping **T** to process the proximity vector **p***_s_* it has stored when it aided anchors exchanging proximity information.
  (4) $${\text{l}}_{s}={\text{Tp}}_{s}$$
  Finally, the node position is calculated by multilateration with the processed proximity vector and the position information of primary and secondary anchors.

Figures 22 - 24 give the pseudo-codes of the actions of primary anchors, normal sensors and secondary anchors in uniform networks.

### 3.3. Computational Complexity

For normal sensors, the number of operations required is comparable to existing distributed protocols. The additional operations compared to other multilateration algorithms is the pre-process of the proximity vector which is a matrix multiplication between the proximity vector and the proximity-distance map.

For secondary anchors, their positions are determined by the classical MDS and they are responsible to calculate the proximity-distance map. Both processes involve the computation of the Singular Value Decomposition (SVD) of a matrix whose complexity is in the order of *O*(*M*^3^) where *M* is the total number of primary and secondary anchors. The additional complexity introduced compared to that of PDM is the adddition of secondary anchors. This increases the matrix size when the proximity-distance map is calculated. However, as simulation results presented in the next section show, the number of secondary anchors required is minimal (around 10-20). The complexity of localizing the secondary anchors by MDS together with determining the proximity-distance map is similar to that of determining a sub-map by a sensor using MDS-MAP(P). The sub-map includes all 2-hop neighbours of a sensor and its size usually exceeds 10 nodes if the average number of neighbours per sensor is about 6.

## 4. Simulation

To justify our proposal, extensive simulations are conducted to study the performance of HyBloc. Simulations are run with MatLab Version 7.2. Each data point is the average result of 30 different simulation runs.

Two hundred nodes are uniformly distributed in a square grid or a C-shaped region. The average connectivity, which is defined as the average number of neighbours per sensor, is controlled by varying the communication range of sensors. Nodes are capable of measuring the distance between itself and any one-hop neighbour (*d̂*). Measurements are subjected to random errors. The error process follows the one adopted in [3], [4] and [8]. The deviation is governed by a normal random process with zero mean and a standard deviation of *α*. This random process is denoted as *N*(0, *α*) and we refer *α* as the noisy factor. That is,

$$\hat{d}=d*(1+N(0,\alpha ))$$

The estimation error is normalized by the communication range *R*. For HyBloc, we randomly pick 20 normal sensors as secondary anchors. We study the effects of anchor placement, connectivity, number of primary anchors, number of secondary anchors and measurement noises. Table 1 gives a summary of the simulation results.

### 4.1. Effects of anchor placement

**Figure 4.**
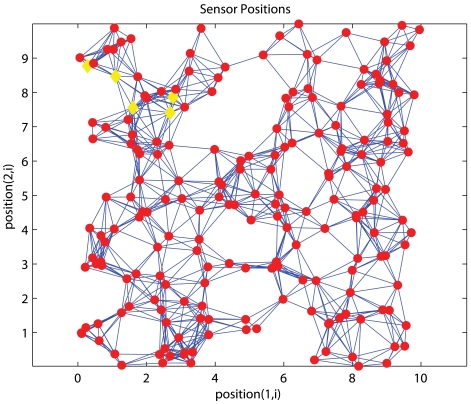
Uniform Network.

**Figure 5.**
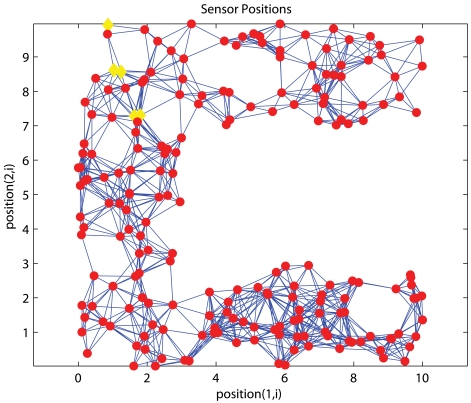
C-shaped Network.

As we previously pointed out that it is not always feasible to deploy anchors across the network, unlike conventional approaches that assume anchors are spread across the network, we study the performance when anchors are confined in a certain part of the network. Figures 4 and 5 show one uniform and C-shaped network respectively where ‘◊’ denotes an anchor.

#### Uniform Networks

**Figure 6.**
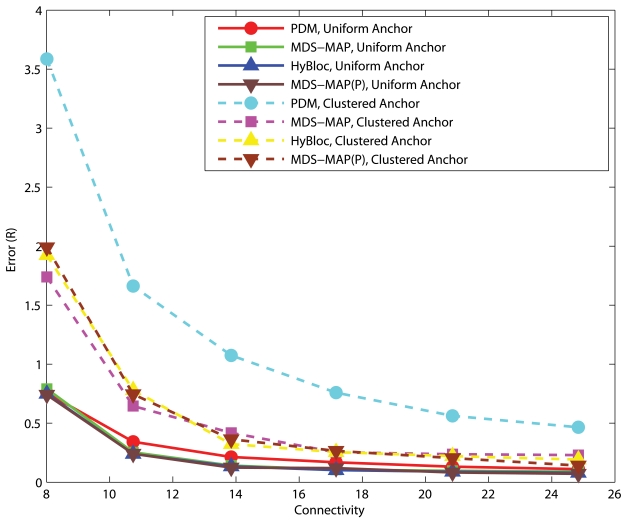
Uniform Networks, *α*=0.05, 5 Anchors.

**Figure 7.**
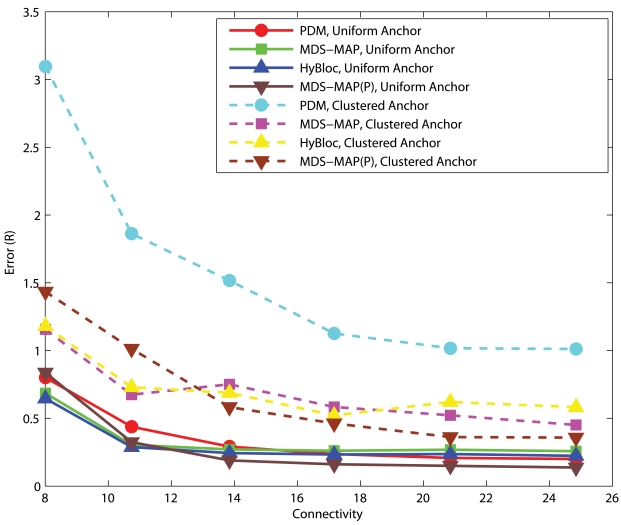
Uniform Networks, *α*=0.10, 5 Anchors.

Figures 6 and 7 show the performance of PDM, HyBloc, MDS-MAP and MDS-MAP(P) where the first two algorithms are distributed algorithms and the latter two are centralized algorithms. Anchors are distributed across the network or clustered together while *α*=0.05 or 0.1. The performance of PDM with clustered anchors is much worse than that of PDM with uniform anchor distribution. With secondary anchors introduced in HyBloc, HyBloc gives a better performance for both scenarios. The average estimation error of HyBloc with 5 clustered anchors and *α*=0.1 is 0.68*R* while the corresponding error of PDM is 1.52*R*, more than twice of the result obtained by HyBloc. The secondary anchors provide a better capture of the relationship between the proximity and geographical distance. MDS-MAP and MDS-MAP(P) give similar result as distance estimates between nodes are relatively accurate in uniform topologies.

#### C-shaped Networks

**Figure 8.**
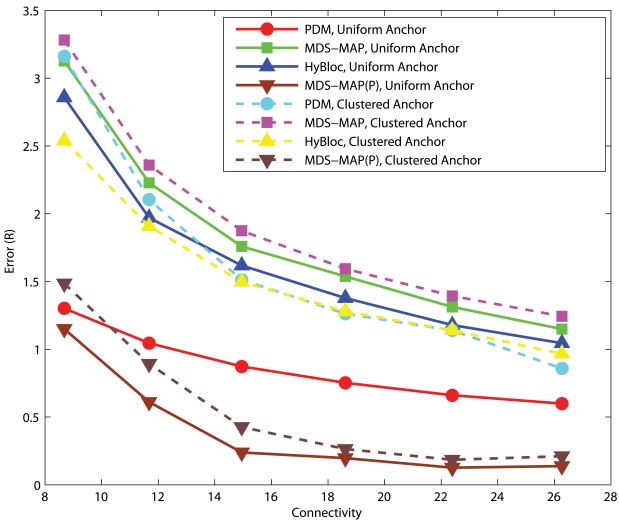
C-shaped Networks, *α*=0.05, 5 Anchors.

**Figure 9.**
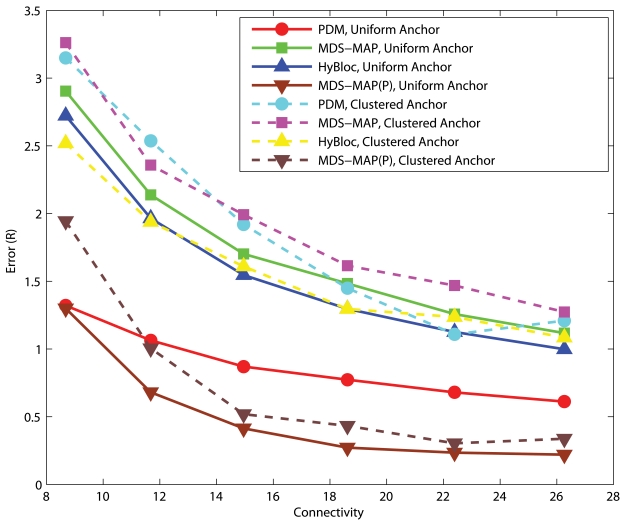
C-shaped Networks, *α*=0.10, 5 Anchors.

Figures 8 and 9 show the corresponding performance in C-shaped networks. HyBloc does not always outperform the other algorithms in C-shaped networks when anchors are uniformly distributed across the networks. PDM even outperforms MDS-MAP and HyBloc with uniform distributed anchors. MDS-MAP(P) yields the best result. Unreliable shortest path distances between distant sensors are avoided by patching a number of local sub-maps to determine the absolute positions. However, the centralized map merging process limits its feasibility in wireless sensor networks. The average error of PDM with connectivity of 14.95 and *α* is equal to 0.1 is 0.87*R* while the corresponding error of HyBloc is about 1.55*R*, almost doubling the error of PDM. The good result from PDM is due to the accurate characterisation of proximity and geographical distance. Furthermore, the sparsity of primary anchors also affects the accuracy of MDS-MAP and the position estimates of secondary anchors. However, HyBloc gives a better performance than PDM when anchors are clustered in a C-shaped network. It is because the position estimates of secondary anchors are much more reliable when primary anchors are clustered and secondary anchors are distributed around the primary anchors. With reliable secondary anchors, better solutions can be obtained in the second phase. If the position estimates of secondary anchors are not reliable enough, it only degrades the overall performance of HyBloc. In summary, HyBloc should be employed when anchors are clustered together or the underlying network is uniform.

### 4.2. Effects of the number of primary anchors

In this section, we will investigate the effects of the number of primary anchors on the performance of PDM, MDS-MAP, MDS-MAP(P) and our proposed HyBloc under different network topologies.

#### Uniform Networks

**Figure 10.**
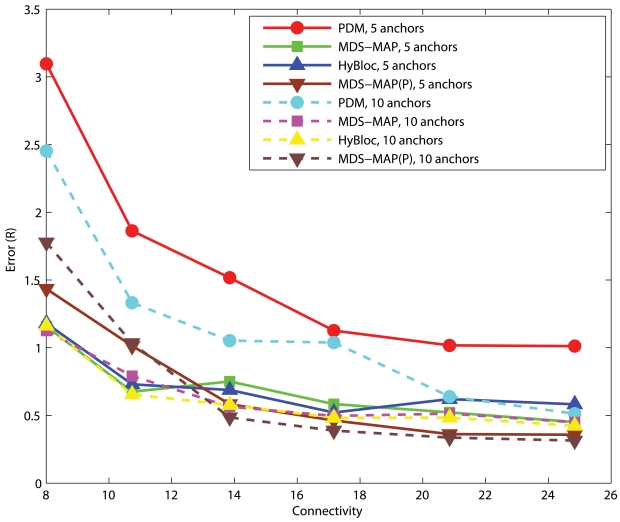
Clustered anchors, *α* = 0.1, Uniform networks.

Figure 10 shows the performance of PDM, MDS-MAP, MDS-MAP(P) and HyBloc with different numbers of clustered anchors under different degrees of connectivity. HyBloc performs consistently better than PDM with 5 or 10 anchors. Though the gap between HyBloc and PDM becomes smaller when connectivity increases to 25 when 10 anchors are available, HyBloc gives a more stable performance. Clearly, adding extra anchors can improve the performance of HyBloc. However, anchors are only used to determine the linear transformation. Thus HyBloc enjoys less benefit from a further increase of anchors and it is less dependent on the number of anchors. For example, the errors given by HyBloc with connectivity = 10.74 and *α*=0.1 are 0.73*R* and 0.66*R* for 5 and 10 anchors, respectively. The corresponding errors produced by PDM are 1.86*R* and 1.3*R*.

#### C-shaped Networks

**Figure 11.**
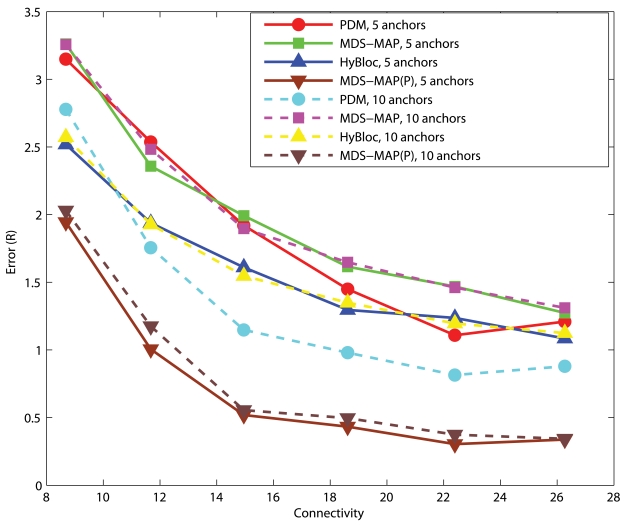
Clustered anchors, *α* = 0.1, C-shaped Networks.

Figure 11 shows the corresponding performance for C-shaped networks. Though HyBloc still gives stable results with different numbers of anchors, PDM provides more accurate solutions when sufficient anchors and large connectivity are available. Similar to spreading anchors across the network, increasing the number of anchors and connectivity imply that more nodes will get involved in determining the transformation between proximity and geographical distance which gives a better solution. On the other hand, by increasing connectivity, primary anchors can reach nodes that are further away. However in C-shaped networks, using path distance as an estimate of the Euclidean distance between nodes that are far apart is very unreliable. It also makes the position estimates in the first phase of HyBloc become unreliable and affects the overall performance of HyBloc when the connectivity is high.

### 4.3. Sensitivity to noise

In this section, the effect of measurement noise will be examined. We test HyBloc and other benchmark algorithms under different noisy factors, ranging from 0 to 0.2.

#### Uniform Networks

**Figure 12.**
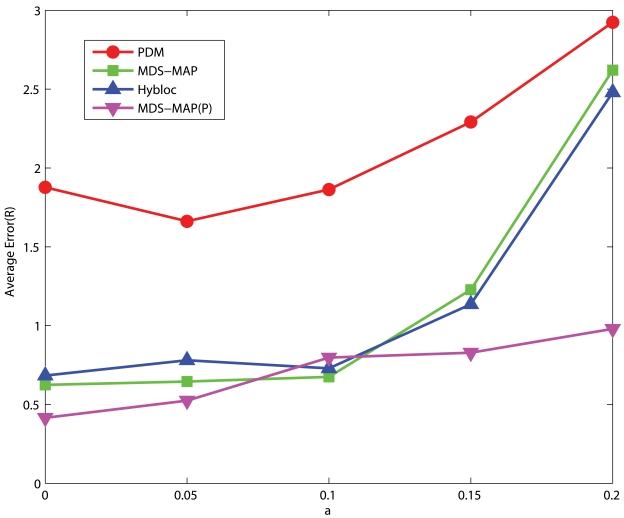
5 Clustered Anchors, Average Connectivity=10.74, Uniform Networks.

**Figure 13.**
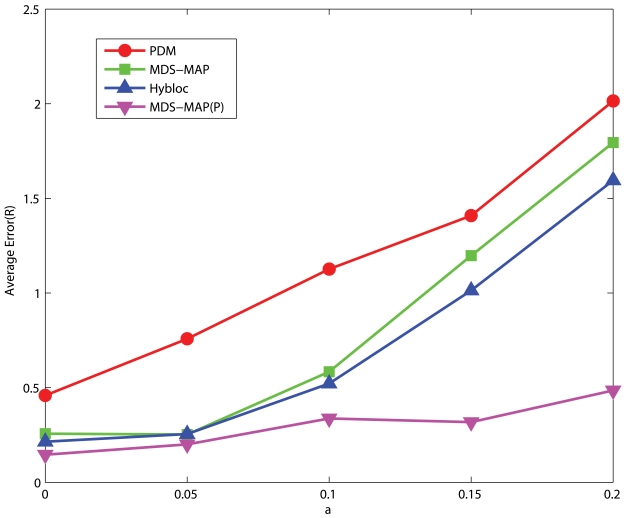
5 Clustered Anchors, Average Connectivity=17.16, Uniform Networks.

Figures 12 and 13 present the performance of networks with low and high connectivity under different degrees of measurement errors. The position estimation error of HyBloc grows significantly when *α* is larger than 0.1. HyBloc gives an accuracy of 1.14*R* when *α* is equal to 0.15 but strikes to 2.4*R* when *α* becomes 0.2. Though the error of HyBloc increases sharply when *α* goes beyond 0.1 for uniform networks with low connectivity, the error of HyBloc is smaller than PDM for all measurement errors considered. For high connectivity, the error rate changes less abruptly than that of low connectivity. The error of HyBloc grows from 1.01*R* to 1.59*R* when *α* increases from 0.15 to 0.2. Furthermore, the performance of HyBloc is still better than that of PDM under all measurement errors considered.

#### C-shaped Networks

**Figure 14.**
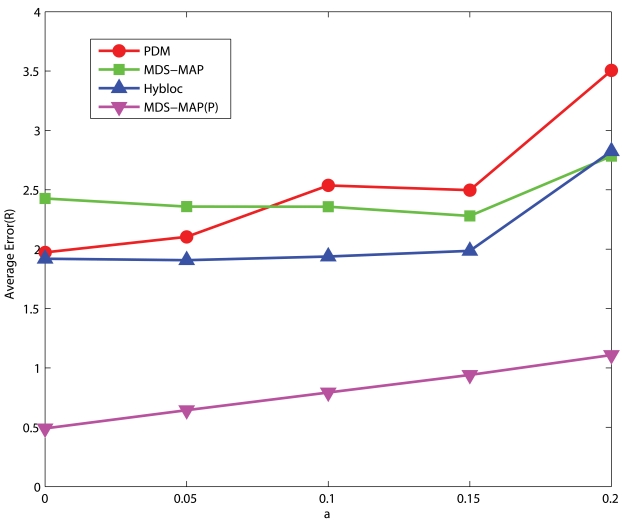
5 Clustered Anchors, Average Connectivity=11.68, C-shaped Networks.

**Figure 15.**
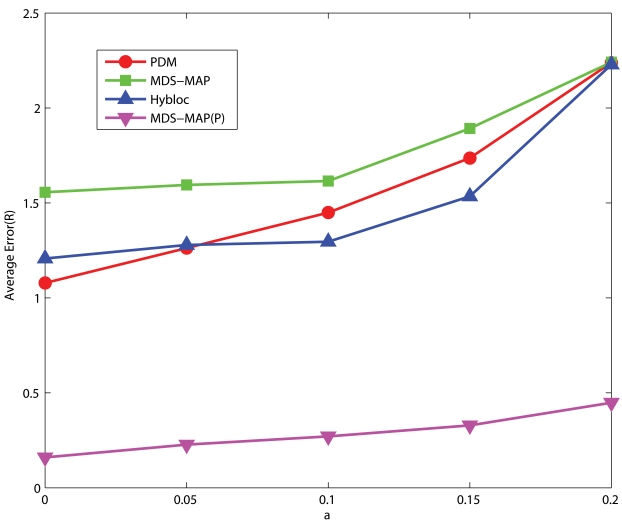
5 Clustered Anchors, Average Connectivity=18.61, C-shaped Networks.

Figures 14 and 15 give the corresponding statistics for performance of HyBloc and PDM in C-shaped networks. Except for the case of high connectivity with exact measurement, HyBloc gives more accurate results than PDM. Unlike the scenarios with uniform networks, the gap between HyBloc and PDM grows as *α* increases. For networks of high connectivity, the average errors of HyBloc and PDM with *α* equals to 0.1 are 1.23*R* and 1.29*R*, respectively. As *α* increases to 0.2, the difference between HyBloc and PDM is more significant. The average error of HyBloc is 2.06*R* while the average error of PDM is 2.42*R*.

### 4.4. Effects of the number of secondary anchors

**Figure 16.**
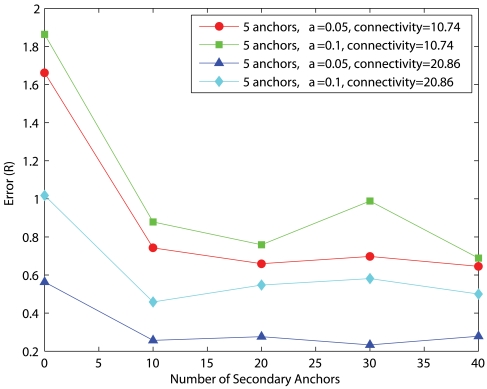
5 Anchors, Uniform Networks.

**Figure 17.**
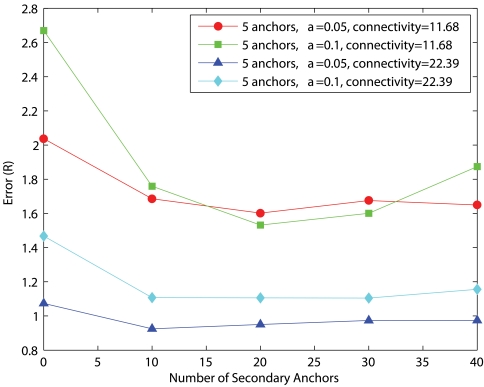
5 Anchors, C-shaped Networks.

After considering the computation and communication costs of MDS, the number of secondary anchors should be minimal to give satisfactory performance. To study the effect of the number of secondary anchors, we vary the number of secondary anchors from 0 (i.e. pure PDM) to 40. Figures 16 and 17 show the corresponding performance for uniform networks and C-shaped networks, respectively. In general, the average estimation error decreases as secondary anchors are introduced but any further increase of secondary anchors beyond 10 does not give any significant improvement. For uniform networks of low connectivity with *α*=0.05, HyBloc gives the average accuracy of 0.74*R* and 0.65*R* when there are 10 and 40 secondary anchors, respectively. The corresponding errors are 1.69*R* and 1.65*R* respectively for C-shaped networks. For high connectivity, the error with *α*=0.05 for uniform networks is 0.25*R* for 10 secondary anchors while the error with 40 secondary anchors is 0.28*R*. The corresponding errors in C-shaped networks are 0.92*R* and 0.97*R* for 10 and 40 secondary anchors, respectively.

In view of the complexity incurred from the first phase of HyBloc and the marginal performance gain through increasing the number of secondary anchors, the number of secondary anchors should be chosen from 10-20 (that is 5% to 10% of the network size).

### 4.5. Effects on the position of anchor cluster

In previous investigations, anchors are clustered in the corner of the network. In this section, we examine the effect of the positions of anchors on the estimation errors.

#### Uniform Networks

For uniform networks, the position of the anchor cluster does not affect the general performance of PDM, MDS-based algorithms nor HyBloc. For PDM, the accuracy is mainly affected by the characterisation of the transformation which is affected by the size of the region covered by the anchors instead of the positions. For MDS-MAP, the performance is also independent of the position of the anchors as the major purpose of anchors is determining the absolute coordinates for the relative map. However, we can anticipate that the performance will be affected in C-shaped networks as path distance becomes an unreliable estimate of the Euclidean distance between two nodes that are far apart.

#### C-shaped Networks

**Figure 18.**
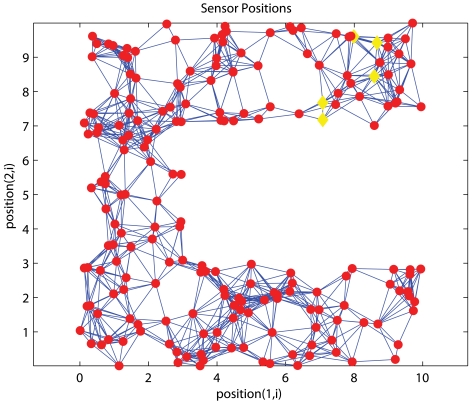
5 Anchors clustered at the tip of ‘C’.

**Figure 19.**
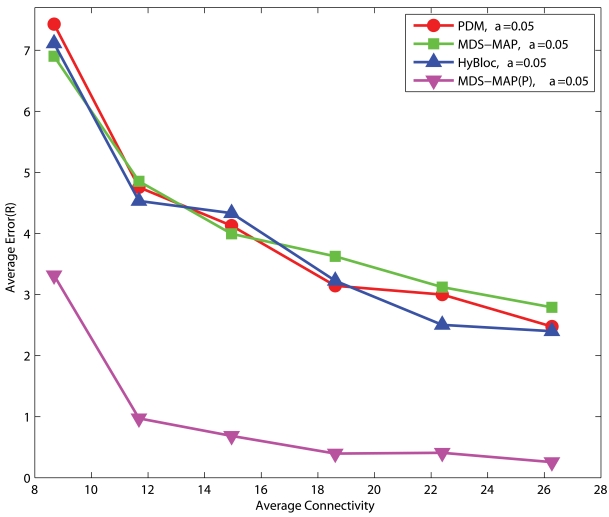
5 Anchors, *α*=0.05, C-shaped Networks.

Instead of putting the cluster of anchors at the top left corner, we placed the anchor cluster at the tip of the ‘C’. Figure 18 shows one specific instance. Figure 19 shows the performance of PDM, MDS-MAP, MDS-MAP(P) and HyBloc with *α*=0.05. By comparing with Figure 8, we can see that the error surges for more than a double except for MDS-MAP(P) when anchors are clustered at the tip of the ‘C’. The accuracy drops drastically as the path connecting most of the nodes and anchors detoured around the ‘C’ which makes the path distance greatly over-estimate the true Euclidean distance. Since MDS-MAP(P) does not depend on the shortest path distance between distant nodes, its error does not rise as others.

### 4.6. Communication Overheads

Since secondary anchors are introduced in the HyBloc algorithm, more messages are transmitted comparing with PDM. The additional messages are incurred by the selection and localization of secondary anchors. Each secondary anchor has to perform a flooding so that all other nodes can estimate the proximities from the secondary anchors. Furthermore, extra messages are also used to determine the location of secondary anchors and the proximity-distance map. It requires the primary and secondary anchors to exchange their proximity vectors to each other. Suppose that there are *x* primary anchors and *y* secondary anchors. Our protocol requires a total of (*x* + *y*)^2^ unicast messages while PDM incurs *x*^2^ unicast messages. In MDS-MAP(P), although nodes determine their local maps which cover neighbours within 2 hops, the map merging process is still centralized in nature. A large amount of messages are needed to collect the sub-maps for map merging and to distribute the position estimates between the sensors and a dedicated node.

**Figure 20.**
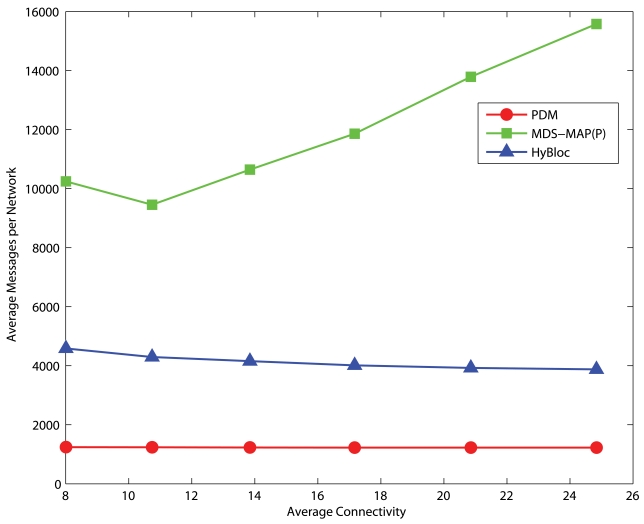
Average number of messages transmitted per uniform network.

**Figure 21.**
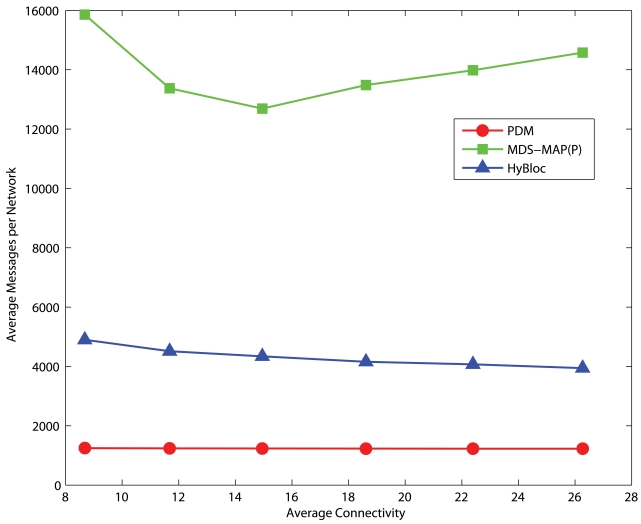
Average number of messages transmitted per C-shaped network.

**Figure 22.**
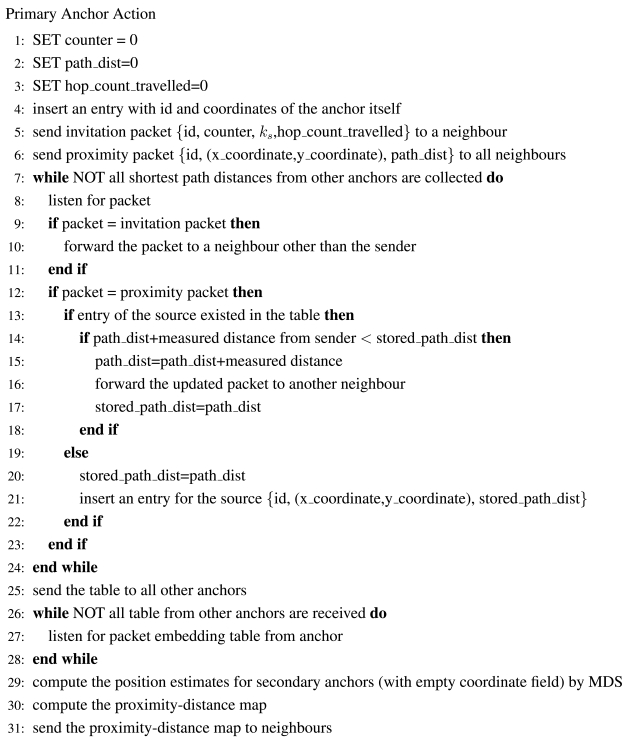
The Pseudo-Code of a Primary Anchor.

**Figure 23.**
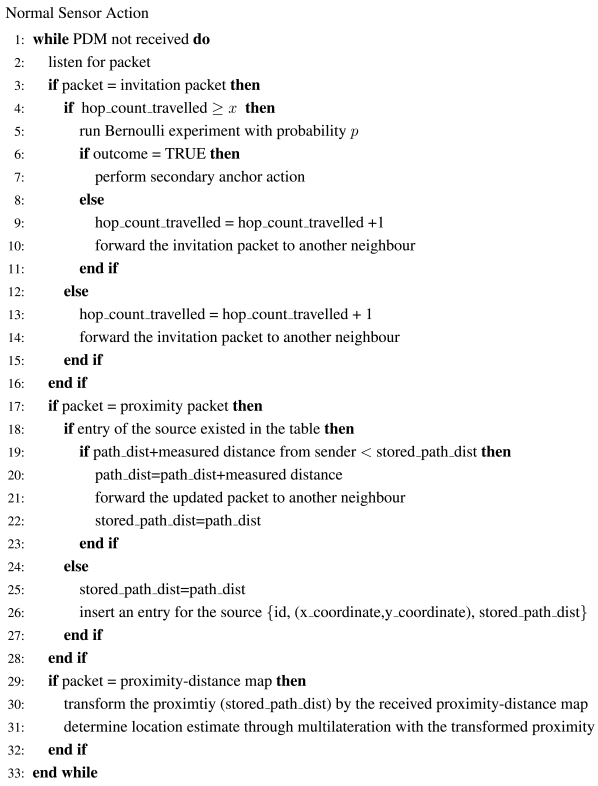
The Pseudo-code of a Normal Sensor.

**Figure 24.**
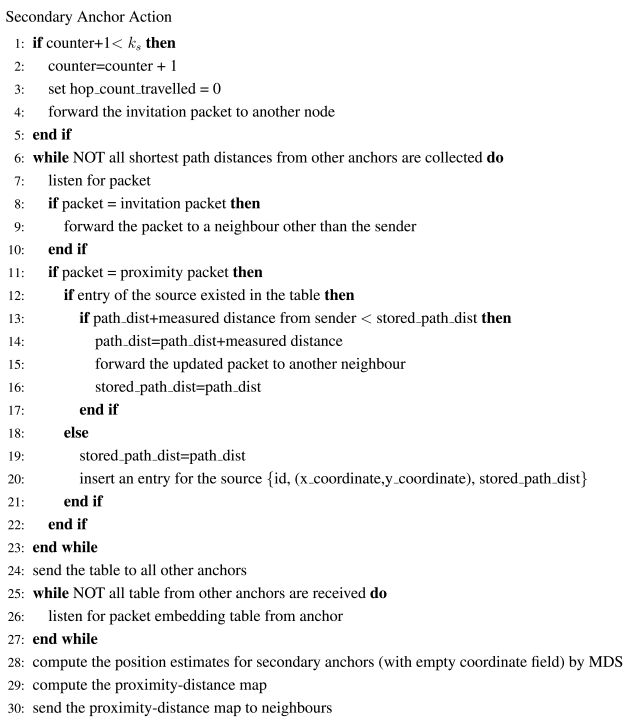
The Pseudo-code of a Secondary Anchor.

Figures 20 and 21 present the average number of messages transmitted in uniform and C-shaped networks, respectively. There are 5 anchors in each network and 10 secondary anchors are selected for HyBloc. Since the map merging process is centralized, the number of messages required by MDS-MAP(P) is several times of that of HyBloc. Also noted that the centralized map merging process also introduces a hot-spot problem in the network. High data traffic appears around the node that determines the global map. On the other hand, although HyBloc requires more messages than PDM because of the extra phase performed by the secondary anchors, the number of messages does not vastly increase with the degrees of connectivity.

## 5. Conclusion

In this paper, we present a hybrid localization algorithm which performs well with very few anchors. Reducing the number of anchors deployed can reduce the cost of sensor network substantially. Through extensive simulations, we demonstrate that the proposed algorithm gives results as accurate as those of MDS-MAP under uniform networks even when the number of anchors is minimal. At the same time, our algorithm exhibits less complexity by employing secondary anchors and PDM in the second phase of localization. Although HyBloc requires more messages than PDM, the message overheads are much smaller than that of MDS-MAP(P). Simulation results also show that our proposal is less susceptible to adverse effect of anchor placement. The proposed algorithm can be implemented in a distributed fashion efficiently when the number of secondary anchors is chosen appropriately. From simulations, we find that choosing secondary anchors from 5% to 10% of network size can give good performance.

However, most networks in real-world applications are not isotropic. For anisotropic networks, though HyBloc gives better results than PDM when anchors are clustered together, there is room for improvement on accuracy. The major difficulty encountered in localization in anisotropic networks is to obtain a reliable distance measurement between two nodes that are far away from each other. PDM devises a transformation between the path distance and the geographical distance between anchors which helps nodes to refine their path distance estimations. Unfortunately, the anchors may not be distributed across the network all the time. The fact that anchors may be placed close to each other puts the reliability of the transformation devised in doubt. To circumvent this problem, we introduce the secondary anchors in order to increase the coverage of anchors. However, as MDS does not perform very well in anisotropic networks, the position estimations of secondary anchors are less accurate than in isotropic networks. In view of this difficult situation, a possible extension is to perform the localization sequentially and introduce secondary anchors gradually outward from the cluster of anchors. In the future, we would like to explore how to perform more accurate localization for C-shaped networks where anchors are clustered in the tip. We also would like to analyse the performance of the various localization mechanisms from an analytical perspective.

## Figures and Tables

**Table 1.** Summary of Simulation Results.

PDM	MDS-MAP, MDS-MAP(P)	HyBloc

Performs well in both isotropic and anisotropic networks with moderate amount of anchors uniformly distributed across the network. Performance deteriorates much when anchors are clustered together. Requires the least amount of messages compared to MDS-MAP(P) and HyBloc.	MDS-MAP performs poorly in anisotropic networks but MDS-MAP(P) performs equally well in different topologies. Performance is less dependent on the number of anchors. Very few anchors (4 to 5) are enough. Centralized in nature, requires a large amount of messages.	Performs well with clustered anchor placement. Increases of primary anchors gives marginal improvement as performance of MDS is less dependent on number of anchors. 5% to 10% of sensors should be chosen as secondary anchors. Performs relatively poorer in C-shaped networks than in uniform networks. Requires moderate amount of messages.
